# Outcomes in Patients with Acute Myocardial Infarction and Known Sleep Apnea: A Nationwide Analysis

**DOI:** 10.3390/jcm12185924

**Published:** 2023-09-12

**Authors:** Claudio Rabec, Chan Sombrun, Sid Ahmed Bentounes, Marjolaine Georges, Arnaud Bisson, Florence Bichat, Alexandre Bodin, Julien Herbert, Marianne Zeller, Yves Cottin, Laurent Fauchier

**Affiliations:** 1 Pneumology Department, CHU Dijon Bourgogne, 21000 Dijon, France; claudio.rabec@chu-dijon.fr (C.R.); marjolaine.georges@chu-dijon.fr (M.G.); 2 Cardiology Department, CHU Dijon Bourgogne, 21000 Dijon, France; chan.sombrun@chu-dijon.fr (C.S.); florence.bichat@chu-dijon.fr (F.B.); marianne.zeller@chu-dijon.fr (M.Z.); 3 Service de Cardiologie, Centre Hospitalier Universitaire Trousseau et Faculté de Médecine, EA7505, Université de Tours, 37020 Tours, France; sidahmed.bentounes@yahoo.fr (S.A.B.); arnaud.bisson37@gmail.com (A.B.); alexandrebodin.mail@gmail.com (A.B.); j.herbert@chu-tours.fr (J.H.); laurent.fauchier@univ-tours.fr (L.F.); 4 Service D’information Médicale, D’épidémiologie et D’économie de la Santé, Centre Hospitalier Universitaire et Faculté de Médecine, EA7505, Université de Tours, 37020 Tours, France; 5 PEC2, EA 7460, UFR Sciences de Santé, Université Bourgogne Franche Comté, 21000 Dijon, France

**Keywords:** atrial fibrillation, myocardial infarction, sleep apnea, death, cardiovascular death, stroke, heart failure, prognosis

## Abstract

**Background**. Sleep apnea (SA) is a common breathing disorder characterized by repetitive upper airway narrowing and closure. Although SA has been demonstrated to be an independent risk factor for all-cause mortality, the direct contribution of SA to worse cardiovascular prognosis may be difficult to evaluate, and its independent association with the different types of cardiovascular outcomes may be debated, particularly in the context of patients with acute myocardial infarction (AMI). The aim of this study was to assess the impact of known SA on the outcomes of hospitalized patients who have had an AMI by analyzing 10-year data collected from a national registry. **Methods**. This longitudinal cohort study was based on the national hospitalization database that covers hospital care for the entire French population, including all patients admitted with AMI from January 2010 to June 2019. The clinical outcomes for the analysis were as follows: all-cause death, cardiovascular death, ischemic stroke, new-onset atrial fibrillation (FA), and re-hospitalization for heart failure (HF). **Results**. Among the 797,212 patients who presented with an AMI (528,351 men and 268,861 women), 37,075 (4.7%) had documented SA. During follow-up (mean [SD] 1.8 [2.4] years, median [interquartile range] 0.7 [0.1–3.1] years), 163,845 deaths (of which 85,649 were cardiovascular deaths), 20,168 ischemic strokes, 58,498 new-onset AF, and 92,381 rehospitalizations due to HF were recorded. Patients with known SA had a worse prognosis in the short and medium term, but after adjusting for all covariables, SA was only independently associated with a higher risk of rehospitalization for HF and new-onset AF in men and women. **Conclusion**. Data from our large nationwide analysis confirm that known SA is associated with poor cardiovascular outcomes in patients who have had an AMI. However, this impact is tem-pered when the model is adjusted for age, cardiovascular risk, or other covariables. Further studies need to be conducted to assess the independent impact of SA on the prognosis of patients with AMI.

## 1. Introduction

Sleep apnea (SA) has been associated with several cardiovascular and metabolic complications. These may include hypertension, atrial fibrillation (AF) and other arrhythmias, heart failure (HF), coronary artery disease, stroke, pulmonary hypertension (PH), metabolic syndrome, and diabetes [1]. Moreover, data from studies have suggested that SA is related to increasing cardiovascular mortality [2,3,4]. Notably, SA is a disorder with potential for negative feedback in which it worsens conditions that may in turn worsen the SA (e.g., SA worsens cardiac failure that may worsen SA). Although SA has been demonstrated to be an independent risk factor for all-cause mortality, the direct contribution of SA to worse cardiovascular prognosis may be difficult to evaluate, and its independent association with the different types of cardiovascular outcomes may be debated, particularly in the context of patients with acute myocardial infarction (AMI) [5]. 

The aim of this longitudinal study based on the national hospitalization database was to assess the short- and medium-term impact of SA on the outcomes of patients hospitalized with AMI by analyzing 10 years of data collected in a national registry.

## 2. Methods

### 2.1. Data Collection

This longitudinal cohort study was based on the national hospitalization database that covers hospital care for the entire French population. All patients admitted with AMI in France from January 2010 to June 2019 were identified using the national administrative PMSI (*Programme de Médicalisation des Systèmes d’Information*) database, which was inspired by the US Medicare system. Through this program, which was implemented in 2004, medical activity is recorded in a database, computed, and rendered anonymous. It covers more than 98% of the French population (67 million people) from birth (or immigration) to death (or emigration), even if a person changes occupation or retires. This process has helped to determine the budget of 1546 French healthcare facilities, including both public and private hospitals. Each hospitalization is encoded in a standardized dataset, which includes information about the patient (age at diagnosis and sex), the hospital, the hospital stay (date of admission, date of discharge, and modes of discharge), pathologies, and procedures. In the PMSI system, the identified diagnoses are coded according to the International Classification of Diseases, Tenth Revision (ICD-10). All medical procedures are recorded according to the national nomenclature, *Classification Commune des Actes Medicaux* (CCAM). The PMSI contains individual anonymized information on each hospitalization, and this information is linked to create a longitudinal record of hospital stays and diagnoses for each patient. The reliability of the PMSI data has already been assessed, and this database has been used to study patients with cardiovascular conditions, including AMI and AF [6,7]. Use of medication was identified from a 1/97 permanent random sample of the complete French nationwide claims database (*Echantillon Généraliste de Bénéficiaires*, EGB—general sample of healthcare beneficiary), which has been used to study patients with acute coronary syndrome in France with the same inclusion criteria as those in the present analysis (patients with AMI) [8,9,10,11]. Patients were considered to be part of a treatment group if they received treatment from that class of drugs for ≥60 days within 6 months of enrolment. 

This study was conducted retrospectively, and as patients were not involved, there was no impact on their care. Ethical approval was not required as all data were anonymized. The French Data Protection Authority granted access to the PMSI data. Procedures for data collection and management were approved by the *Commission Nationale de l’Informatique et des Libertés*, the independent National Ethical Committee protecting human rights in France, which ensures that all information is kept confidential and anonymous, in compliance with the Declaration of Helsinki (authorization number 1897139). 

### 2.2. Study Population

From January 2010 to June 2019, a total of 797,212 patients aged 18 years or older were hospitalized with an AMI (ICD-10 codes: I21, I22, and I23) as the principal (i.e., the health problem that justified admission to hospital), the related (i.e., potential chronic disease or health state during the hospital stay), or a significantly associated (i.e., comorbidity or associated complication) diagnosis. Information was collected on demographics, medical history, procedures, and events during hospitalization or follow-up. SA was also identified with its specific ICD code (G473). The exclusion criterion was age < 18 years.

### 2.3. Outcomes

Patients were followed up until June 2019 for the incidence of outcomes, starting from the date of the first report of AMI in the database. The endpoints were evaluated with follow-up, starting with the date of each specific outcome or the date of the last record in the absence of the outcome. The clinical outcomes for the analysis were as follows: all-cause death, cardiovascular death, and hospitalization for HF and ischemic stroke. Information on outcomes during the follow-up was obtained by analyzing the PMSI codes for each patient. All-cause death, cardiovascular death, ischemic stroke, and HF were identified using their respective ICD-10 codes. Hospitalization was considered to be related to HF if HF was recorded as the main diagnosis. The mode of death (cardiovascular or non-cardiovascular) was identified based on the main diagnosis during hospitalization resulting in in-hospital death. 

### 2.4. Statistical Analyses 

Qualitative variables are described as frequency and percentages and quantitative variables as means ± standard deviation (SD). Incidence rates for outcomes in each group were estimated, and cumulative incidence curves were obtained using the Kaplan–Meier method. To obtain adjusted hazard ratios (HR), a multivariate analysis of clinical outcomes during the whole follow-up period for the groups of interest was performed using a Cox model with relevant baseline characteristics, namely, age, sex, Charlson comorbidity index, frailty index, obesity, dyslipidemia, diabetes mellitus, hypertension, smoking, alcohol-related diagnoses, previously known coronary artery disease, previous percutaneous coronary intervention (PCI), dilated cardiomyopathy, previous HF, previous ischemic stroke, previous pacemaker or defibrillator, vascular disease, abnormal renal function, liver disease, lung disease, chronic obstructive pulmonary disease (COPD), SA, anemia, previous cancer, history of metastasis, thyroid diseases, cognitive impairment, depression, denutrition, ST-segment elevation myocardial infarction (STEMI), non-ST-segment elevation myocardial infarction (NSTEMI), anterior AMI, and inferior AMI (Supplemental Figure S1). A *p* value < 0.05 was considered statistically significant. All analyses were performed using Enterprise Guide 7.1 (SA Institute Inc., SA Campus Drive, Cary, NC, USA) and STATA version 16.0 (Stata Corp., College Station, TX, USA).

## 3. Results

**Figure 1:**
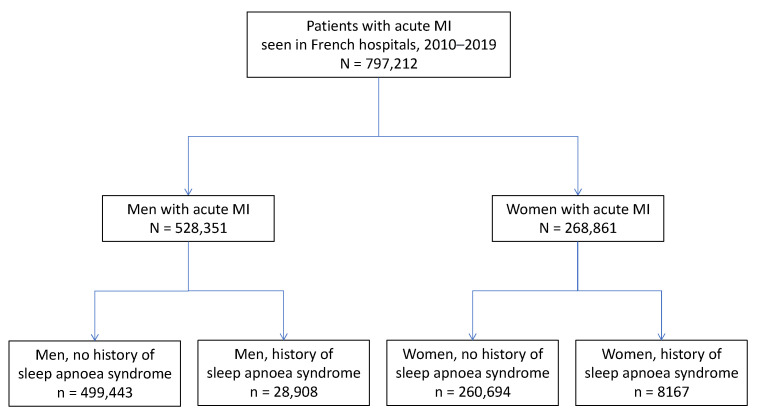
Flowchart of the study population.

Among the 797,212 patients who presented with an AMI (528,351 men and 268,861 women), 37,075 (4.7%) had documented SA. A history of SA was more prevalent in men than in women (5.5% vs. 3.0%, *p* < 0.0001) (Figure 1). Men with documented SA were older than those without SA, while women with documented SA were younger than those without. Patients with known SA had a higher prevalence of most cardiovascular and non-cardiovascular comorbidities (Table 1). However, they had less frequent STEMI and anterior AMI. In addition, they underwent PCI less frequently but had more frequent coronary artery bypass graft than other patients. 

**Figure 2:**
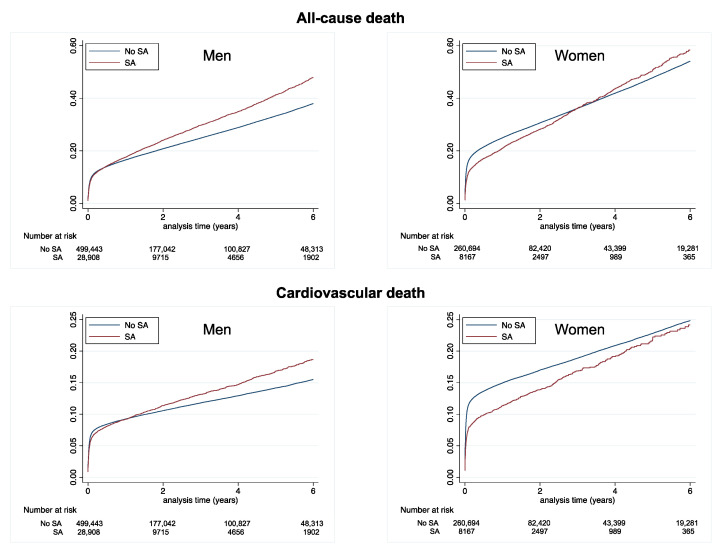
Cumulative incidence of all-cause death (top panels) and cardiovascular death (lower panels) in men (left panels) and women (right panels) with AMI according to a history of SA or no SA.

**Figure 3:**
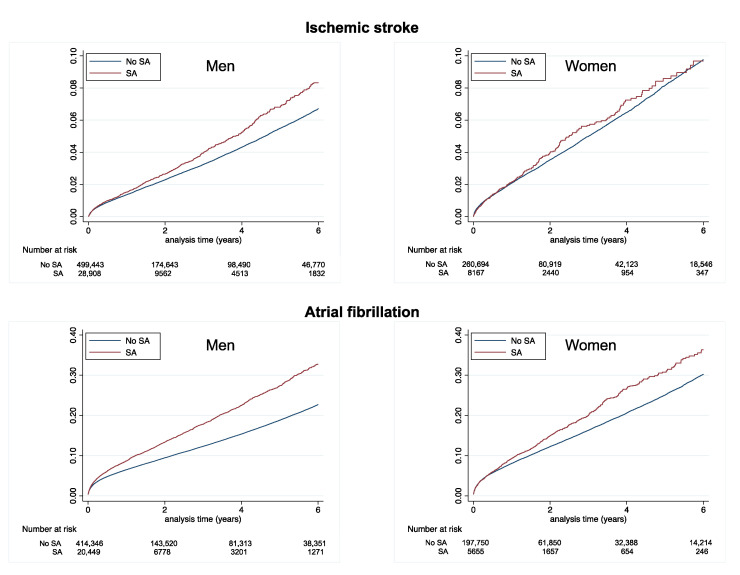
Cumulative incidence of hospitalization for heart failure (top panels) and ischemic stroke (lower panels) in men (left panels) and women (right panels) with AMI according to a history of SA or no SA.

**Figure 4:**
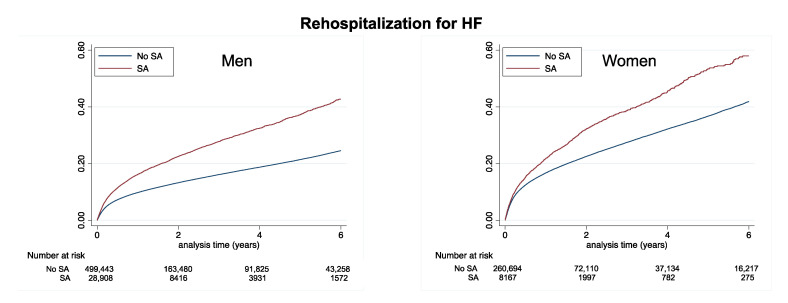
Cumulative incidence of new-onset AF in men (left panels) and women (right panels) with AMI according to a history of SA or no SA.

During follow-up (mean [SD] 1.8 [2.4] years, median [interquartile range] 0.7 [0.1–3.1] years), 163,845 deaths (of which 85,649 were cardiovascular deaths), 20,168 ischemic strokes, 58,498 new-onset AF cases, and 92,381 rehospitalizations due to HF were recorded. The cumulative incidences of these events in patients with AMI, according to previous SA, are displayed in Table 2 and in Figure 2 for all-cause death and cardiovascular death, in Figure 3 for ischemic stroke and new-onset AF, and in Figure 4 for rehospitalization for HF. In these unadjusted analyses, patients with no SA had the best survival and the lowest risk of ischemic stroke (Table 2 and Table 3).

The results of the multivariable analyses are presented in Table 3, along with age-adjusted analysis (model B), adjustment for cardiovascular risk factors (model C), and adjustment for all of the covariables presented in Table 1 other than the cardiovascular risk factors (model D). In the age-adjusted analysis, SA was associated with a higher risk of all-cause (but not cardiovascular) death, rehospitalization for HF, ischemic stroke, and new-onset AF in both groups of male and female patients. In the model adjusted for cardiovascular risk factors, SA was associated with a higher risk of all-cause (but not cardiovascular) death, rehospitalization for HF, and new-onset AF in both groups of male and female patients (Supplementary Tables S1 and S2). In the fully adjusted model, SA was independently associated with only a lower risk of all-cause death and cardiovascular death in men (Table 3). In addition, in the fully adjusted model, SA was only independently associated with a higher risk of rehospitalization for HF and with new-onset AF in men and women (Table 3).

## 4. Discussion

In this large nationwide study, patients with known SA and AMI had a worse prognosis in the short and medium term, but after adjusting for all covariables, SA was only independently associated with a higher risk of rehospitalization for HF and new-onset AF in men and women.

Our results are in line with data obtained from three large cohort studies evaluating the association of SA and cardiovascular morbimortality. The Sleep Heart Health Study cohort, evaluating 6424 patients, demonstrated that moderate-to-severe SA is associated with an increased risk of 1.27 for all cardiovascular events [1]. On the other hand, The Wisconsin Sleep Cohort study, which included 1522 patients, reported an HR of 2.4 for incident ischemic heart disease (IHD) and an odds ratio (OR) of 4.5 for stroke in patients with severe SA [12]. Finally, a study by Marin and colleagues, conducted in Spain and including 1949 patients, demonstrated that untreated SA significantly increased the risk of nonfatal cardiovascular events (OR, 3.71) [13].

The specific relationship between SA and IHD has also been addressed in several studies. It has been suggested that patients with SA have a greater prevalence of IHD (with a dose–effect relationship), an increased incidence of coronary artery disease independent of other risk factors, and a higher rate of recurrent ischemic events and cardiovascular mortality [13,14,15,16,17]. However, a meta-analysis published in 2012 showed that SA was a significant risk factor for IHD only in men and not in women (OR, 1.92; 95% CI, 1.06–3.48) [18].

In our univariate analysis, SA had an impact on cardiovascular mortality, but this effect was lost when the model was adjusted for age. Moreover, when we applied the model adjusted for cardiovascular risk factors, we failed to find any impact on cardiovascular mortality, while the association with all-cause mortality remained. One can assume that, as in other studies, the presence of cardiovascular disease at baseline was the strongest predictor of cardiovascular death, neutralizing any additional risk related to SA [2]. 

A fully adjusted model integrating all variables failed to demonstrate a significant independent impact of SA on cardiovascular or on all-cause mortality. 

This is in opposition to the results of other large cohort studies. The Wisconsin Sleep Cohort study reported an adjusted OR for moderate–severe SA of 1.4–3.8 and 1.2–5.2 for all-cause and cardiovascular mortality, respectively [12]. On the other hand, the Busselton Health Study found an adjusted HR of 4.2 for all-cause mortality [19]. Finally, in Marin’s study, severe SA significantly increased the risk of fatal cardiovascular events with an OR of 2·87, 95%CI 1.17–7.51 [2]. This difference could be explained by the different variables used in the adjusted model. In the Wisconsin study, the risk was adjusted only for age, sex, and body mass index; in the Busselton Health study, it was adjusted for age, gender, obesity, smoking status, blood pressure, dyslipidemia, angina, cancer history, and diabetes, and in the Spanish study, the risk was adjusted by taking into account variables with a significant unadjusted association. Another possible explanation is the potential bias due to the effect of SA treatment. Indeed, in our study, we had no data on the rate of patients treated with continuous positive airway pressure (CPAP), a first-line therapy for moderate-to-severe SA. A preliminary prospective longitudinal study suggested that CPAP treatment could reduce cardiovascular morbimortality in patients with SA [20]. However, a recent randomized controlled study demonstrated that CPAP did not prevent cardiovascular events in patients with moderate-to-severe SA and established cardiovascular disease. Moreover, for an estimated population of 11 million people with moderate-to-severe SA in France, only approximately 1,200,000 (11%) receive CPAP [21]. In this regard, the potential confounding impact of patients on CPAP in our cohort seems to be slight.

Our study found a difference in the role of gender on the impact of SA on some outcomes. This was not unexpected. First, it has been reported that SA is underdiagnosed in women. They present with more “atypical” symptoms, but even when presenting with a standard clinical picture, women with SA are significantly less likely to be diagnosed than men despite equal risks of developing hypertension and diabetes [22]. Second, female patients have shorter event durations and lower desaturation compared to male patients with similar apnea–hypopnea index (AHI) values. This could explain the lower cardiovascular impact for the same AHI [23]. Finally, sex-specific differences exist in the relationship between SA and cardiovascular disease [24]. For example, a cohort study demonstrated that stroke is more prevalent in men than in women with SA [24]. This is in line with our unadjusted results. 

### 4.1. Preexisting SA and Acute Coronary Syndrome

Our study included only patients with known SA and an acute coronary syndrome. Indeed, many studies have emphasized the importance of systematic screening after acute coronary syndrome. The work carried out by Fan and colleagues (published in 2019) regarding a cohort of 833 patients showed a prevalence of SA of 50%, defined as an AHI of >15 events h^−1^ [25]. 

Comparable results were found in a study published in 2022 by Wang. Among 2028 patients, 298 (15.5%) women and 1014 (52.6%) men had SA (AHI ≥ 15 events·h^−1^) [26]. Moreover, SA was associated with a greater risk of a major adverse cardiovascular and cerebrovascular event (MACCE) in women (28.1% vs. 18.8%) but not in men (21.6% vs. 17.5%) [15]. In addition, the incremental risk in women was attributable to higher rates of hospitalization for unstable angina and ischemia-driven revascularization.

Acute MI patients with recognized SA had significantly decreased mortality compared with patients without SA [27, 28]. On the basis of a real healthcare system, our results confirm that of previous studies that show the cardioprotective association of SA over the medium and long term after MI, but only in men. There are several explanations for these findings. Firstly, the “obesity paradox” is a well-known phenomenon that has shown that obese patients with established cardiovascular disease have a better prognosis [27,28,29,30]. The obesity paradox can be explained by earlier symptoms, more aggressive optimization of medical treatment, but also neurohormonal alterations such as increased soluble tumor necrosis factor-alpha receptors [27,28,29,30]. The second most likely mechanism is acquired pre-conditioning from nightly intermittent hypoxic episodes in patients with SA, resulting in the development of collaterals and therefore less extensive infarcts [31]. Thirdly, intermittent hypoxia in patients with AS is associated with an increase in hypoxia-inducible factor concentration, which leads to a better response to hypoxia and increases cell survival [27,31]. 

### 4.2. Screening of SA in Acute Coronary Syndrome

The data from our study are in agreement with that of the literature, as evidenced by our study showing a percentage of AS known on admission for Acute MI of 4.5%, close to that of a recent work by Mohananey D and colleagues, which showed a percentage of 1.5% [28]. However, numerous studies show that the systematic screening of patients with coronary artery disease is associated with an AS prevalence of 40–50% [32].

A prospective cohort study published by Mooe and colleagues in 2001 included 408 patients aged 70 years or younger with verified coronary disease who were followed up for a median period of 5.1 years [33]. An AHI of ≥10 and an oxygen desaturation index (ODI) of ≥5 were used as the diagnostic criteria for sleep-disordered breathing. The primary endpoint was a composite of death, cerebrovascular events, and MI. There was a 70% relative increase and a 10.7% absolute increase in the primary composite endpoint (death, cerebrovascular events, and MI) in patients with disordered breathing (defined as an ODI of ≥5). Similarly, patients with an AHI of ≥10 had a 62% relative increase and a 10.1% absolute increase in the composite endpoint. In this work, however, the results were not adjusted for factors associated with excess risk after acute coronary syndrome.

A recent multicenter, randomized controlled trial included patients with acute coronary syndrome from 15 hospitals in Spain [34]. All patients underwent respiratory polygraphy during the first 24–72 h after admission. Patients with SA were randomly assigned (1:1) to CPAP treatment plus usual care (CPAP group) or usual care alone (UC group) through a computerized system. The rate of SA was 49.55% for a median of 3.35 years of follow-up; the prevalence of cardiovascular events was similar in the CPAP and UC groups, and the prevalence of cardiovascular events did not seem to be related to CPAP compliance or SA severity [34]. 

In addition, principal component analysis of the results from the ISAACC study showed that only long-duration obstructive respiratory events without severe desaturation were associated with an increased risk of recurrent cardiovascular events in patients with SA and acute coronary syndrome [34].

### 4.3. Atrial Fibrillation and SA Following Myocardial Infarction

Obesity is a risk factor for AF, and SA is highly prevalent in obesity [14,35]. Gami and colleagues conducted a retrospective cohort study of 3542 Olmsted County adults without past or current AF who were referred for initial diagnostic polysomnography from 1987 to 2003 [36]. New-onset AF was assessed and confirmed by electrocardiography during a mean follow-up of 4.7 years [36]. These authors demonstrated that in patients younger than 65 years, independent predictors of incident AF were age, male gender, coronary artery disease, body mass index (per 1 kg/m^2^, HR, 1.07; 95% CI, 1.05–1.10), and the decrease in nocturnal oxygen saturation. By contrast, HF, but neither obesity nor SA, predicted incident AF in patients ≥65 years of age.

In recent decades, there has been increasing evidence demonstrating that SA is associated with the development of AF [37]. Furthermore, SA was strongly associated with a high risk of incident AF and a higher recurrence rate of AF after initially successful cardioversion [37,38]. Moreover, SA patients with SA show slower response rates to antiarrhythmic drugs [39]. 

Whether this association reflects a causal effect is still unclear, as SA and AF are two common diseases in adults. However, recently, Chen and coworkers suggested a causal effect of genetically predicted SA on the risk of AF [40].

There were several assumed mechanisms underlying AF that were attributable to SA. Repetitive obstructive respiratory events may cause substantially negative intrathoracic pressure that leads to structural remodeling through repetitive mechanical atrial distension and atrial wall stretch. Moreover, frequent episodes of hemoglobin desaturation and resaturation result in the inflammation and hyperactivity of the cardiac autonomic nervous system, which may be implicated in atrial arrhythmogenesis [41].

In our study, new-onset AF was the only unfavorable outcome associated with SA that remained both in men and in women when the model was adjusted for all covariables. Moreover, new-onset AF and rehospitalization are the outcomes most impacted by SA, showing higher HRs.

### 4.4. SA and Heart Failure Following Myocardial Infarction

Despite advances in cardiac care, patients who have recently experienced MI are at a high risk of developing chronic HF and of death; even with current therapies, between 12% and 17% of patients are at risk of death or hospitalization for HF in the 12–22 months following MI [42,43,44]. These risks are the highest in the 10–38% of patients with reduced ejection fraction or signs and symptoms of HF following MI, who are at a greater than two-fold increased risk of death following MI [45,46,47,48]. In addition, data from the SWEDEHEART registry identified the following strong predictors of late-onset heart failure (LOHF) after MI: (a) in-hospital HF, (b) age, (c) diabetes mellitus, (d) chronic kidney disease, (e) peripheral arterial disease, (f) chronic obstructive pulmonary disease, and (g) AF [43]. 

Sleep apnea is independently associated with an increased risk of adverse outcomes, including HF-related symptom progression, hospitalization, and mortality. Patients without HF diagnosed with SA have an increased subsequent risk of incident HF [1,35]. The pathophysiological effects of SA relevant to HF are mediated by several mechanisms, including neurohormonal activation, increased oxidative stress and inflammation, acute increases in preload and afterload related to large intrathoracic pressure swings, and the exacerbation of systemic hypertension [1,35]. In our study SA was independently associated with a higher risk of rehospitalization for HF, but this effect was verified only in men (not in women) when the model was adjusted for all covariables.

### 4.5. Limitations

Aside from the retrospective, observational nature of this study and its potential biases, it was also based on administrative data obtained and manually completed by physicians and administrators. Data were not externally checked systematically, and this could have caused information bias. However, as coding is linked to reimbursement and is regularly controlled, it is expected that the data are of good quality. Analyses were restricted to the variables coded in the database, excluding the precise results of coronary angiograms and echocardiographic measurements and changes in antithrombotic therapy. Only in-hospital events were included, and we were not able to analyze data for out-of-hospital deaths, but most of the major cardiovascular events analyzed in our study did not take place outside of hospitals. Further, the observational design of our analysis leaves a risk of residual confounding factors. Comparisons between groups might not be fully appropriate, as possible confounding factors cannot be ruled out. It is possible that SA may have been more actively monitored and diagnosed in some subgroups of patients (those with obesity or with symptoms suggestive of SA). Our large population of hospitalized patients most likely represents a heterogeneous group of patients admitted with various kinds of illnesses and severities, which may have affected the prognosis. Finally, the majority of the French population is white, and our results may not be generalizable to non-white people.

## 5. Conclusions

The data from our large nationwide analysis confirm that SA is associated with poor cardiovascular outcomes in patients who have had an AMI. However, this impact is tempered when the model is adjusted for age, cardiovascular risk, or other covariables. In a model adjusted for all relevant covariables, SA remained independently associated with a higher risk of rehospitalization for HF in men and of new-onset AF both in men and women. Further studies need to be conducted to assess the independent impact of SA in the prognosis of patients with MI. 

## Figures and Tables

**Table 1:** Characteristics of patients with acute myocardial infarction (AMI).

			Men			Women	
	Total	No Sleep Apnea Syndrome	Sleep Apnea Syndrome	*p*	No Sleep Apnea Syndrome	Sleep Apnea Syndrome	*p*
	(*n* = 797,212)	(*n* = 499,443)	(*n* = 28,908)		(*n* = 260,694)	(*n* = 8167)	
Age (years), mean ± SD	69.1 ± 14.9	65.8 ± 14.3	68.2 ± 11.7	<0.0001	75.5 ± 14.3	72.4 ± 11.7	<0.0001
Sex (male), *n* (%)	528,351 (66.3)	499,443 (100.0)	28,908 (100.0)	-	0 (0.0)	0 (0.0)	-
Charlson comorbidity index, mean ± SD	3.8 ± 2.9	3.6 ± 2.9	5.2 ± 3.4	<0.0001	3.9 ± 2.7	5.7 ± 3.3	<0.0001
Frailty index, mean ± SD	6.2 ± 8.1	5.1 ± 7.3	7.6 ± 8.6	<0.0001	8.0 ± 8.9	10.0 ± 9.6	<0.0001
Obesity, *n* (%)	132,312 (16.6)	72,554 (14.5)	15,222 (52.7)	<0.0001	39,261 (15.1)	5275 (64.6)	<0.0001
Dyslipidemia, *n* (%)	267,840 (33.6)	169,163 (33.9)	15,694 (54.3)	<0.0001	78,662 (30.2)	4321 (52.9)	<0.0001
Diabetes mellitus, *n* (%)	192,456 (24.1)	110,902 (22.2)	13,276 (45.9)	<0.0001	63,771 (24.5)	4507 (55.2)	<0.0001
Hypertension, *n* (%)	440,432 (55.2)	246,164 (49.3)	22,404 (77.5)	<0.0001	164,801 (63.2)	7063 (86.5)	<0.0001
Smoker, *n* (%)	173,728 (21.8)	132,245 (26.5)	8398 (29.1)	<0.0001	31,802 (12.2)	1283 (15.7)	<0.0001
Alcohol-related diagnoses, *n* (%)	42,217 (5.3)	32,860 (6.6)	2889 (10.0)	<0.0001	6170 (2.4)	298 (3.6)	<0.0001
Coronary artery disease, *n* (%)	167,855 (21.1)	103,965 (20.8)	12,663 (43.8)	<0.0001	48,093 (18.4)	3134 (38.4)	<0.0001
Previous PCI, *n* (%)	38,369 (4.8)	24,993 (5.0)	3427 (11.9)	<0.0001	9189 (3.5)	760 (9.3)	<0.0001
Dilated cardiomyopathy, *n* (%)	43,528 (5.5)	23,898 (4.8)	3052 (10.6)	<0.0001	15,612 (6.0)	966 (11.8)	<0.0001
Heart failure with congestion, *n* (%)	82,752 (10.4)	40,088 (8.0)	6498 (22.5)	<0.0001	33,695 (12.9)	2471 (30.3)	<0.0001
Ischemic stroke, *n* (%)	30,326 (3.8)	15,993 (3.2)	1631 (5.6)	<0.0001	12,192 (4.7)	510 (6.2)	<0.0001
Atrial fibrillation, *n* (%)	146,922 (18.4)	78,378 (15.7)	8252 (28.5)	<0.0001	57,870 (22.2)	2422 (29.7)	<0.0001
Previous pacemaker or ICD, *n* (%)	25,346 (3.2)	14,923 (3.0)	2364 (8.2)	<0.0001	7552 (2.9)	507 (6.2)	<0.0001
Vascular disease, *n* (%)	103,933 (13.0)	64,816 (13.0)	8743 (30.2)	<0.0001	28,350 (10.9)	2024 (24.8)	<0.0001
Abnormal renal function, *n* (%)	53,847 (6.8)	28,237 (5.7)	4077 (14.1)	<0.0001	20,066 (7.7)	1467 (18.0)	<0.0001
Liver disease, *n* (%)	26,589 (3.3)	16,242 (3.3)	1984 (6.9)	<0.0001	7748 (3.0)	615 (7.5)	<0.0001
Lung disease, *n* (%)	107,049 (13.4)	61,142 (12.2)	9776 (33.8)	<0.0001	32,897 (12.6)	3234 (39.6)	<0.0001
COPD, *n* (%)	61,950 (7.8)	37,275 (7.5)	7142 (24.7)	<0.0001	15,658 (6.0)	1875 (23.0)	<0.0001
Anemia, *n* (%)	92,307 (11.6)	45,950 (9.2)	4790 (16.6)	<0.0001	39,531 (15.2)	2036 (24.9)	<0.0001
Previous cancer, *n* (%)	87,988 (11.0)	56,631 (11.3)	4715 (16.3)	<0.0001	25,664 (9.8)	978 (12.0)	<0.0001
History of metastasis, *n* (%)	16,432 (2.1)	9885 (2.0)	701 (2.4)	<0.0001	5658 (2.2)	188 (2.3)	0.42
Thyroid diseases, *n* (%)	52,495 (6.6)	15,039 (3.0)	2104 (7.3)	<0.0001	33,358 (12.8)	1994 (24.4)	<0.0001
HIV infection, *n* (%)	2521 (0.3)	2119 (0.4)	73 (0.3)	<0.0001	322 (0.1)	7 (0.1)	0.34
Illicit drug use, *n* (%)	4328 (0.5)	3596 (0.7)	139 (0.5)	<0.0001	561 (0.2)	32 (0.4)	0.001
Cognitive impairment, *n* (%)	54,422 (6.8)	22,118 (4.4)	1923 (6.7)	<0.0001	29,471 (11.3)	910 (11.1)	0.65
Depression, *n* (%)	58,668 (7.4)	23,209 (4.6)	3030 (10.5)	<0.0001	30,443 (11.7)	1986 (24.3)	<0.0001
Poor nutrition, *n* (%)	48,781 (6.1)	21,696 (4.3)	1943 (6.7)	<0.0001	24,180 (9.3)	962 (11.8)	<0.0001
STEMI, *n* (%)	520,258 (65.3)	332,503 (66.6)	15,425 (53.4)	<0.0001	168,133 (64.5)	4197 (51.4)	<0.0001
NSTEMI, *n* (%)	276,954 (34.7)	166,940 (33.4)	13,483 (46.6)	<0.0001	92,561 (35.5)	3970 (48.6)	<0.0001
Anterior MI, *n* (%)	221,344 (27.8)	140,515 (28.1)	6216 (21.5)	<0.0001	72,884 (28.0)	1729 (21.2)	<0.0001
Inferior MI, *n* (%)	173,162 (21.7)	119,107 (23.8)	5180 (17.9)	<0.0001	47,700 (18.3)	1175 (14.4)	<0.0001
MI with other location, *n* (%)	402,706 (50.5)	239,821 (48.0)	17,512 (60.6)	<0.0001	140,110 (53.7)	5263 (64.4)	<0.0001
HF at the acute phase, *n* (%)	199,601 (25.0)	117,246 (23.5)	5564 (19.2)	<0.0001	75,083 (28.8)	1708 (20.9)	<0.0001
Pulm. edema/shock at the acute phase, *n* (%)	48,129 (6.0)	28,426 (5.7)	1532 (5.3)	0.01	17,703 (6.8)	468 (5.7)	0.0002
PCI at the acute phase, *n* (%)	241,387 (30.3)	175,495 (35.1)	7096 (24.5)	<0.0001	57,412 (22.0)	1384 (16.9)	<0.0001
PCI during first 8 days, *n* (%)	423,932 (53.2)	298,199 (59.7)	14,176 (49.0)	<0.0001	108,414 (41.6)	3143 (38.5)	<0.0001
CABG at the acute phase, *n* (%)	14,723 (1.8)	10,873 (2.2)	790 (2.7)	<0.0001	2961 (1.1)	99 (1.2)	0.52
CABG during first 8 days, *n* (%)	29,880 (3.7)	22,316 (4.5)	1494 (5.2)	<0.0001	5864 (2.2)	206 (2.5)	0.1
Death at 1 month, *n* (%)	78,826 (9.9)	41,005 (8.2)	2271 (7.9)	0.03	34,742 (13.3)	808 (9.9)	<0.0001
Cardiovascular death at 1 month, *n* (%)	56,582 (7.1)	29,083 (5.8)	1531 (5.3)	0.0002	25,421 (9.8)	547 (6.7)	<0.0001

Values are mean ± standard deviation or *n* (%). CABG = coronary artery bypass graft; MI = myocardial infarction; PCI = percutaneous coronary intervention; SD = standard deviation.

**Table 2:** Incidences of clinical outcomes during the whole follow-up period (mean [SD] 1.8 [2.3], median [IQR] 0.7 [0.1–3.1] years) in the cohort of men and women with AMI.

		Men			Women	
Incidence, %/Year (95% CI)	No SA	SA	*p*	No SA	SA	*p*
All-cause death	9.39 (9.33–9.45)	11.45 (11.16–11.75)	<0.0001	14.88 (14.76–14.99)	14.65 (14.00–15.32)	0.51
Cardiovascular death	4.68 (4.63–4.72)	5.39 (5.20–5.60)	<0.0001	8.32 (8.24–8.41)	7.31 (6.86–7.79)	0.0001
Ischemic stroke	1.21 (1.18–1.23)	1.42 (1.32–1.53)	<0.0001	1.80 (1.76–1.84)	1.89 (1.67–2.15)	0.42
New-onset AF	4.56 (4.52–4.61)	6.52 (6.26–6.79)	<0.0001	6.04 (5.96–6.13)	7.42 (6.87–8.01)	<0.0001
Rehospitalization for HF	5.42 (5.38–5.47)	9.97 (9.68–10.27)	<0.0001	9.72 (9.63–9.82)	14.59 (13.88–15.32)	<0.0001

Values are *n* (incidence rate, %/year). CI = confidence interval; HF = heart failure; PY = patient-year; SA = sleep apnea syndrome.

**Table 3:** Hazard ratios (HR) for clinical outcomes during the whole follow-up period (mean [SD] 1.8 [2.3], median [IQR] 0.7 [0.1–3.1] years) in the cohorts of men and women with AMI with SA or no SA.

HR (95% CI)	Model A	Model B	Model C	Model D
Men				
All-cause death	1.142 (1.112–1.172)	1.121 (1.092–1.151)	1.092 (1.063–1.123)	0.901 (0.876–0.926)
Cardiovascular death	1.055 (1.015–1.096)	1.029 (0.990–1.069)	1.037 (0.997–1.079)	0.923 (0.887–0.961)
Ischemic stroke	1.183 (1.097–1.276)	1.149 (1.066–1.239)	1.053 (0.974–1.138)	0.978 (0.904–1.059)
New-onset AF	1.393 (1.336–1.453)	1.374 (1.318–1.433)	1.169 (1.119–1.221)	1.059 (1.013–1.107)
Rehospitalization for HF	1.684 (1.633–1.736)	1.659 (1.609–1.710)	1.296 (1.255–1.338)	1.038 (1.004–1.072)
Women				
All-cause death	0.916 (0.875–0.958)	1.146 (1.095–1.200)	1.111 (1.059–1.164)	0.967 (0.922–1.014)
Cardiovascular death	0.804 (0.754–0.858)	1.018 (0.954–1.086)	1.025 (0.959–1.095)	0.985 (0.921–1.053)
Ischemic stroke	1.046 (0.919–1.189)	1.219 (1.071–1.387)	1.105 (0.968–1.262)	1.071 (0.937–1.225)
New-onset AF	1.191 (1.101–1.288)	1.479 (1.367–1.600)	1.256 (1.158–1.362)	1.147 (1.057–1.245)
Rehospitalization for HF	1.351 (1.284–1.421)	1.695 (1.611–1.783)	1.244 (1.180–1.310)	1.030 (0.977–1.086)

Model A: unadjusted. Model B: age-adjusted Model C: adjusted for cardiovascular risk factors from Table 1 Model D: adjusted for all covariables in Table 1 *. CI = confidence interval; HF = heart failure; HR = hazard ratio; * Adjusted for age, sex, Charlson comorbidity index, frailty index, obesity, dyslipidemia, diabetes mellitus, hypertension, smoking, alcohol-related diagnoses, previously known coronary artery disease, previous PCI, dilated cardiomyopathy, previous heart failure, previous ischemic stroke, previous pacemaker or defibrillator, vascular disease, abnormal renal function, liver disease, lung disease, COPD, sleep apnea syndrome, anemia, previous cancer, history of metastasis, thyroid diseases, cognitive impairment, depression, denutrition, STEMI, NSTEMI, anterior MI, inferior MI.

## Data Availability

The data and study materials will not be made available to other researchers for the purposes of reproducing the results or replicating the procedure. Because this study used data from human subjects, the data and everything pertaining to the data are governed by the French Health Agencies and cannot be made available to other researchers.

## Supplementary Materials

The following supporting information can be downloaded at: https://www.mdpi.com/article/10.3390/jcm12185924/s1, Table S1: Hazard ratios (HR) for clinical outcomes using multivariable analysis from model D in the cohorts of men with AMI with SA or no SA; Table S2: Hazard ratios (HR) for clinical outcomes using multivariable analysis from model D in the cohorts of women with AMI with SA or no SA; Figure S1: Directed acyclic graph (DAG) derived from literature and medical knowledge.
