# Supplementary material for: Outcomes in Patients with Acute Myocardial Infarction and Known Sleep Apnea: A Nationwide Analysis
Source: J Clin Med. 2023 Sep 12;12(18):5924. doi: 10.3390/jcm12185924 (PMC10532263; doi:10.3390/jcm12185924)
Supplement: Supplementary file 1 [file jcm-12-05924-s001.zip › jcm-2536951-supplementary.pdf]

Supplemental Table S1. Hazard ratios (HR) for clinical outcomes using multivariable analysis from model D in the cohorts of men with AMI with SA or no SA.

	All-cause death	Cardiovascular death	Ischemic stroke	New-onset AF	Rehospitalization for HF
	**HR, 95%CI**	**HR, 95%CI**	**HR, 95%CI**	**HR, 95%CI**	**HR, 95%CI**
**Age (years)**	1.048 (1.048-1.049)	1.050 (1.049-1.051)	1.015 (1.013-1.017)	1.039 (1.038-1.040)	1.037 (1.036-1.038)
**Frailty index**	1.011 (1.010-1.012)	0.992 (0.991-0.993)	1.039 (1.037-1.041)	1.023 (1.021-1.024)	1.013 (1.012-1.014)
**Charlson comorbidity index**	1.008 (1.005-1.011)	0.915 (0.911-0.920)	1.227 (1.219-1.235)	1.091 (1.086-1.096)	1.133 (1.128-1.137)
**Heart failure with congestion**	1.739 (1.711-1.767)	2.315 (2.261-2.370)	0.958 (0.916-1.002)	1.326 (1.293-1.361)	2.678 (2.624-2.733)
**History of pulmonary edema**	3.002 (2.948-3.056)	3.514 (3.431-3.599)	1.066 (0.980-1.159)	1.085 (1.033-1.141)	1.184 (1.150-1.220)
**Coronary artery disease**	0.997 (0.980-1.015)	1.014 (0.988-1.040)	0.970 (0.921-1.022)	1.036 (1.006-1.068)	1.113 (1.088-1.139)
**Previous PCI**	0.946 (0.921-0.973)	0.915 (0.879-0.952)	1.049 (0.967-1.137)	0.938 (0.895-0.983)	0.947 (0.916-0.978)
**Previous CABG**	1.085 (1.055-1.116)	1.128 (1.083-1.175)	1.035 (0.947-1.131)	1.031 (0.977-1.087)	1.120 (1.082-1.159)
**Mitral regurgitation**	0.875 (0.854-0.898)	0.909 (0.877-0.941)	1.037 (0.962-1.118)	1.173 (1.124-1.225)	1.156 (1.122-1.190)
**Aortic regurgitation**	0.937 (0.902-0.973)	1.000 (0.948-1.054)	1.028 (0.918-1.151)	1.058 (0.989-1.132)	0.980 (0.937-1.026)
**Dilated cardiomyopathy**	0.954 (0.932-0.977)	0.961 (0.929-0.994)	1.030 (0.956-1.108)	1.161 (1.112-1.212)	1.351 (1.315-1.388)
**Hypertension**	0.910 (0.897-0.924)	0.922 (0.903-0.942)	1.107 (1.061-1.155)	1.124 (1.098-1.151)	1.115 (1.091-1.139)
**Diabetes mellitus**	1.101 (1.084-1.119)	1.320 (1.291-1.350)	0.745 (0.713-0.778)	0.882 (0.859-0.904)	1.063 (1.042-1.085)
**Ischemic stroke**	1.310 (1.275-1.345)	1.773 (1.711-1.838)	-	0.960 (0.907-1.015)	0.836 (0.803-0.871)
**Atrial fibrillation**	1.057 (1.042-1.073)	1.038 (1.016-1.060)	1.140 (1.089-1.193)	1.000 (0.000-0.000)	1.335 (1.309-1.361)
**Previous pacemaker or ICD**	0.986 (0.960-1.012)	1.016 (0.979-1.055)	0.939 (0.857-1.028)	1.161 (1.101-1.225)	1.206 (1.169-1.245)
**Obesity**	0.901 (0.884-0.918)	0.933 (0.908-0.959)	0.895 (0.850-0.942)	1.206 (1.173-1.241)	1.059 (1.035-1.084)
**Dyslipidaemia**	0.777 (0.766-0.788)	0.773 (0.756-0.789)	0.948 (0.911-0.986)	0.919 (0.898-0.940)	0.893 (0.876-0.910)
**Smoker**	0.931 (0.915-0.948)	0.851 (0.829-0.874)	0.916 (0.873-0.961)	0.890 (0.866-0.915)	0.998 (0.975-1.022)
**Vascular disease**	1.195 (1.174-1.217)	1.368 (1.332-1.405)	1.041 (0.986-1.101)	1.040 (1.007-1.074)	0.987 (0.964-1.011)
**Abnormal renal function**	1.143 (1.120-1.166)	1.344 (1.304-1.384)	0.754 (0.701-0.811)	0.924 (0.887-0.963)	1.025 (0.998-1.053)
**Liver disease**	1.647 (1.605-1.691)	1.894 (1.825-1.965)	0.663 (0.595-0.738)	0.834 (0.782-0.890)	0.803 (0.768-0.840)
**Lung disease**	1.355 (1.324-1.386)	1.442 (1.397-1.488)	0.697 (0.638-0.761)	0.896 (0.854-0.940)	0.905 (0.874-0.938)
**COPD**	0.982 (0.957-1.009)	0.892 (0.858-0.926)	1.068 (0.963-1.183)	1.203 (1.138-1.272)	1.155 (1.109-1.202)
**Anaemia**	1.181 (1.160-1.201)	1.045 (1.018-1.073)	0.843 (0.791-0.897)	0.954 (0.920-0.989)	0.944 (0.920-0.968)
**Previous cancer**	1.279 (1.255-1.303)	1.149 (1.116-1.182)	0.700 (0.659-0.743)	0.849 (0.821-0.878)	0.764 (0.744-0.785)
**History of metastasis**	2.315 (2.245-2.388)	1.740 (1.641-1.844)	0.740 (0.632-0.867)	0.773 (0.705-0.848)	0.733 (0.683-0.786)
**Alcohol related diagnoses**	1.311 (1.280-1.343)	1.290 (1.244-1.337)	1.059 (0.983-1.140)	1.113 (1.064-1.164)	1.134 (1.096-1.174)
**Thyroid diseases**	0.955 (0.927-0.983)	0.954 (0.913-0.996)	0.947 (0.860-1.043)	1.044 (0.984-1.107)	1.009 (0.972-1.048)
**HIV infection**	0.931 (0.832-1.042)	1.489 (1.251-1.772)	0.288 (0.217-0.382)	0.492 (0.408-0.593)	0.454 (0.387-0.533)
**Cognitive impairment**	1.228 (1.199-1.256)	1.318 (1.274-1.363)	0.879 (0.805-0.960)	0.704 (0.666-0.744)	0.826 (0.796-0.857)
**Depression**	0.984 (0.959-1.009)	0.974 (0.938-1.012)	1.003 (0.924-1.089)	0.881 (0.838-0.926)	0.948 (0.914-0.983)
**Poor nutrition**	1.055 (1.031-1.079)	0.937 (0.905-0.971)	0.982 (0.896-1.076)	0.849 (0.803-0.897)	0.944 (0.911-0.977)
**STEMI**	1.587 (1.560-1.613)	1.813 (1.769-1.858)	0.920 (0.877-0.967)	0.910 (0.885-0.936)	1.008 (0.985-1.030)
**NSTEMI**	1.000 (0.000-0.000)	1.000 (0.000-0.000)	1.000 (0.000-0.000)	1.000 (0.000-0.000)	1.000 (0.000-0.000)
**Anterior MI**	0.833 (0.820-0.847)	0.898 (0.877-0.919)	1.107 (1.053-1.164)	0.905 (0.879-0.932)	1.122 (1.096-1.149)
**Inferior MI**	0.695 (0.681-0.709)	0.719 (0.700-0.740)	0.959 (0.907-1.014)	0.905 (0.877-0.934)	0.833 (0.810-0.857)
**Sleep apnoea syndrome**	0.901 (0.876-0.926)	0.923 (0.887-0.961)	0.978 (0.904-1.059)	1.059 (1.013-1.107)	1.038 (1.004-1.072)

Supplemental Table S2. Hazard ratios (HR) for clinical outcomes using multivariable analysis from model D in the cohorts of women with AMI with SA or no SA.

	All-cause death	Cardiovascular death	Ischemic stroke	New-onset AF	Rehospitalization for HF
	**HR, 95%CI**	**HR, 95%CI**	**HR, 95%CI**	**HR, 95%CI**	**HR, 95%CI**
**Age (years)**	1.050 (1.049-1.051)	1.055 (1.054-1.056)	1.023 (1.020-1.025)	1.040 (1.039-1.042)	1.039 (1.037-1.040)
**Frailty index**	0.997 (0.996-0.998)	0.976 (0.974-0.977)	1.028 (1.026-1.030)	1.020 (1.019-1.022)	1.009 (1.008-1.010)
**Charlson comorbidity index**	0.952 (0.947-0.956)	0.895 (0.889-0.901)	1.293 (1.282-1.304)	1.106 (1.099-1.113)	1.118 (1.112-1.123)
**Heart failure with congestion**	1.643 (1.613-1.674)	1.893 (1.846-1.942)	0.849 (0.807-0.894)	1.241 (1.203-1.281)	2.413 (2.357-2.470)

**History of pulmonary edema**	3.034 (2.970-3.099)	3.238 (3.152-3.327)	0.991 (0.892-1.101)	1.063 (0.994-1.137)	1.101 (1.061-1.143)
**Coronary artery disease**	0.962 (0.941-0.984)	0.997 (0.967-1.027)	0.884 (0.828-0.944)	0.996 (0.956-1.038)	1.077 (1.048-1.106)
**Previous PCI**	0.909 (0.870-0.949)	0.883 (0.832-0.938)	1.069 (0.948-1.207)	1.044 (0.969-1.125)	0.920 (0.877-0.966)
**Previous CABG**	1.160 (1.101-1.223)	1.202 (1.121-1.290)	1.030 (0.877-1.210)	1.078 (0.971-1.197)	1.162 (1.096-1.233)
**Mitral regurgitation**	0.902 (0.877-0.927)	0.970 (0.935-1.006)	1.015 (0.935-1.101)	1.186 (1.126-1.249)	1.186 (1.149-1.224)
**Aortic regurgitation**	0.971 (0.930-1.015)	1.004 (0.947-1.064)	0.942 (0.828-1.073)	0.980 (0.901-1.065)	1.021 (0.972-1.074)
**Dilated cardiomyopathy**	0.901 (0.875-0.929)	0.889 (0.853-0.926)	0.978 (0.894-1.069)	1.047 (0.988-1.109)	1.128 (1.090-1.166)
**Hypertension**	0.877 (0.862-0.893)	0.878 (0.857-0.899)	1.032 (0.978-1.090)	1.106 (1.070-1.144)	1.119 (1.091-1.148)
**Diabetes mellitus**	1.339 (1.314-1.365)	1.518 (1.479-1.557)	0.670 (0.634-0.708)	0.863 (0.833-0.895)	1.115 (1.088-1.142)
**Ischemic stroke**	1.517 (1.472-1.564)	1.947 (1.873-2.024)	-	0.935 (0.869-1.007)	0.816 (0.779-0.854)
**Atrial fibrillation**	1.087 (1.069-1.106)	1.062 (1.037-1.086)	1.395 (1.326-1.467)	1.000 (0.000-0.000)	1.326 (1.297-1.355)
**Previous pacemaker or ICD**	1.047 (1.008-1.087)	1.051 (0.998-1.106)	1.016 (0.897-1.150)	1.102 (1.011-1.202)	1.209 (1.157-1.263)
**Obesity**	0.953 (0.931-0.976)	0.971 (0.941-1.003)	0.877 (0.821-0.935)	1.102 (1.059-1.147)	1.099 (1.069-1.129)
**Dyslipidaemia**	0.779 (0.765-0.793)	0.790 (0.770-0.810)	0.963 (0.916-1.012)	0.914 (0.886-0.943)	0.894 (0.874-0.914)
**Smoker**	0.971 (0.938-1.006)	0.868 (0.825-0.913)	0.942 (0.858-1.035)	0.831 (0.783-0.883)	0.976 (0.934-1.021)
**Vascular disease**	1.291 (1.260-1.323)	1.464 (1.416-1.513)	0.956 (0.888-1.029)	1.009 (0.962-1.058)	1.003 (0.973-1.034)
**Abnormal renal function**	1.303 (1.271-1.336)	1.413 (1.366-1.463)	0.847 (0.781-0.919)	0.975 (0.925-1.028)	1.048 (1.016-1.081)
**Liver disease**	1.717 (1.656-1.780)	1.833 (1.745-1.926)	0.671 (0.580-0.776)	0.910 (0.827-1.002)	0.892 (0.842-0.946)
**Lung disease**	1.295 (1.261-1.329)	1.275 (1.229-1.323)	0.688 (0.625-0.757)	0.904 (0.852-0.958)	0.927 (0.893-0.962)
**COPD**	1.006 (0.971-1.043)	0.924 (0.877-0.973)	0.912 (0.800-1.041)	1.146 (1.061-1.238)	1.114 (1.061-1.170)
**Anaemia**	1.137 (1.114-1.159)	1.040 (1.012-1.070)	0.872 (0.817-0.930)	0.929 (0.891-0.968)	0.980 (0.954-1.006)
**Previous cancer**	1.354 (1.318-1.391)	1.182 (1.136-1.229)	0.584 (0.534-0.640)	0.839 (0.794-0.886)	0.808 (0.778-0.838)
**History of metastasis**	2.389 (2.283-2.501)	1.588 (1.462-1.725)	0.630 (0.512-0.776)	0.749 (0.658-0.852)	0.711 (0.649-0.778)
**Alcohol related diagnoses**	1.315 (1.251-1.383)	1.249 (1.158-1.347)	1.002 (0.856-1.174)	1.026 (0.923-1.142)	0.995 (0.924-1.071)
**Thyroid diseases**	0.937 (0.916-0.958)	0.944 (0.916-0.973)	0.982 (0.920-1.048)	0.999 (0.957-1.042)	0.970 (0.943-0.997)
**HIV infection**	1.748 (1.336-2.287)	2.557 (1.695-3.858)	0.236 (0.118-0.474)	0.224 (0.107-0.471)	0.578 (0.398-0.838)
**Cognitive impairment**	1.356 (1.326-1.387)	1.436 (1.393-1.480)	0.932 (0.864-1.005)	0.688 (0.653-0.726)	0.813 (0.786-0.841)
**Depression**	0.995 (0.973-1.019)	0.995 (0.963-1.027)	0.969 (0.903-1.041)	0.887 (0.847-0.929)	0.991 (0.962-1.022)
**Poor nutrition**	1.016 (0.993-1.041)	0.937 (0.906-0.969)	1.029 (0.948-1.118)	0.914 (0.865-0.965)	0.976 (0.944-1.009)
**STEMI**	1.659 (1.626-1.694)	1.828 (1.777-1.881)	0.926 (0.874-0.982)	0.887 (0.855-0.919)	1.001 (0.976-1.028)
**NSTEMI**	1.000 (0.000-0.000)	1.000 (0.000-0.000)	1.000 (0.000-0.000)	1.000 (0.000-0.000)	1.000 (0.000-0.000)
**Anterior MI**	0.848 (0.832-0.864)	0.942 (0.918-0.967)	0.966 (0.909-1.026)	0.907 (0.873-0.943)	1.047 (1.019-1.075)
**Inferior MI**	0.775 (0.757-0.793)	0.898 (0.870-0.926)	0.962 (0.898-1.030)	0.941 (0.901-0.983)	0.938 (0.908-0.969)
**Sleep apnoea syndrome**	0.967 (0.922-1.014)	0.985 (0.921-1.053)	1.071 (0.937-1.225)	1.147 (1.057-1.245)	1.030 (0.977-1.086)

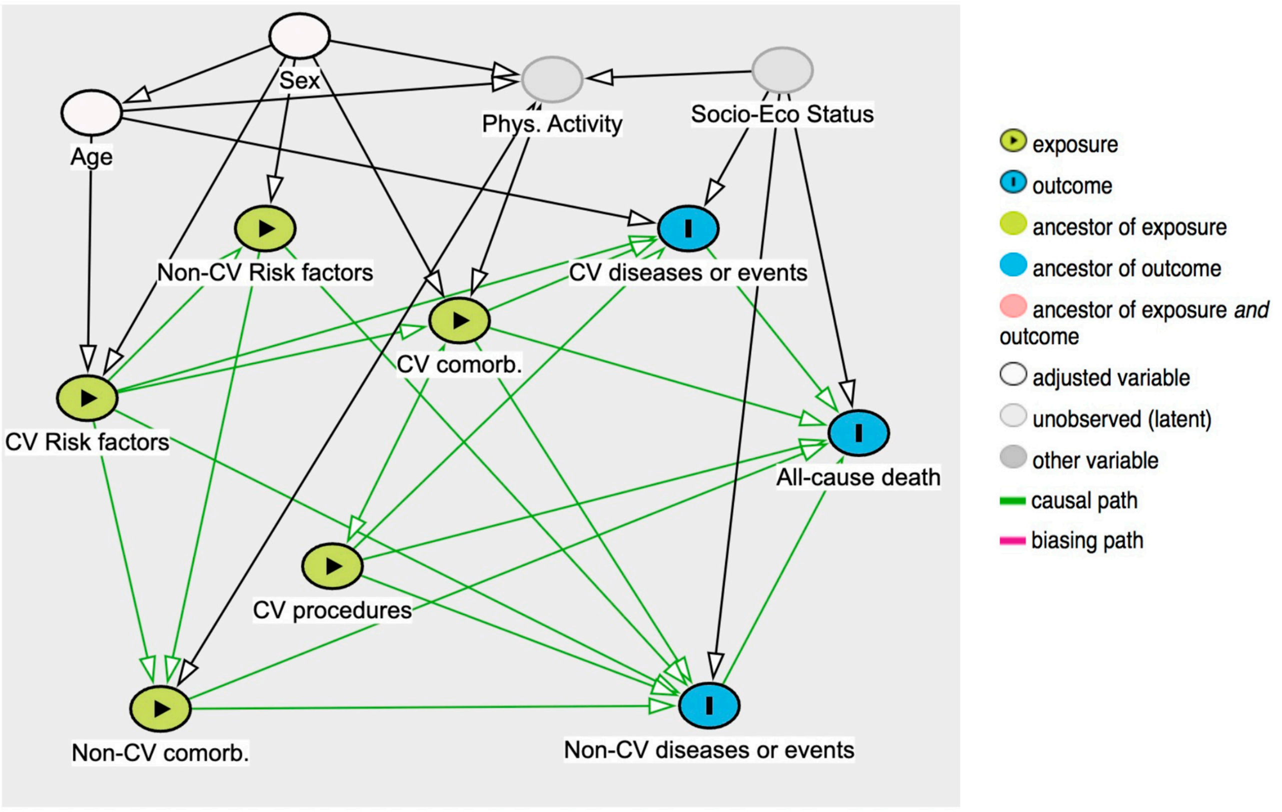

Supplemental Figure S1. Directed acyclic graph (DAG) derived from literature and medical knowledge.

Nodes represent variables and arrows represent causal associations. Green-colored nodes represent exposure and blue-colored node represents outcomes. White-colored nodes represent possible confounding factors and those in grey-colored were not accessible in the present analysis. This simplified version of DAG was used to visually depict assumptions about causal relationships. Possible pairs of variables were assessed applying suspected, known and/or published evidence of causal relationships. Each node represented a variable (or group of variables) of interest. Arrows between nodes denoted causal relationships, pointing from cause to effect, and included even weak assumptions of causal relationships or causal relationships present only in sub-groups. Lack of an arrow denoted that no obvious or major causal relationship exists. There was no ancestor of exposure and outcome in red not taken into account in this DAG for the fully adjusted model.
